# Bioinformatic identification of cassava miRNAs differentially expressed in response to infection by Xanthomonas axonopodis pv. manihotis

**DOI:** 10.1186/1471-2229-12-29

**Published:** 2012-02-23

**Authors:** Álvaro L Pérez-Quintero, Andrés Quintero, Oscar Urrego, Pablo Vanegas, Camilo López

**Affiliations:** 1 Biology Department, Universidad Nacional de Colombia, Calle 45 Cra 30, Bogota, Bogota DC, Colombia

## Abstract

**Background:**

microRNAs (miRNAs) are short RNA molecules that control gene expression by silencing complementary mRNA. They play a crucial role in stress response in plants, including biotic stress. Some miRNAs are known to respond to bacterial infection in *Arabidopsis thaliana *but it is currently unknown whether these responses are conserved in other plants and whether novel species-specific miRNAs could have a role in defense.

**Results:**

This work addresses the role of miRNAs in the *Manihot esculenta *(cassava)-*Xanthomonas axonopodis *pv. manihotis *(Xam) *interaction. Next-generation sequencing was used for analyzing small RNA libraries from cassava tissue infected and non-infected with *Xam*. A full repertoire of cassava miRNAs was characterized, which included 56 conserved families and 12 novel cassava-specific families. Endogenous targets were predicted in the cassava genome for many miRNA families. Some miRNA families' expression was increased in response to bacterial infection, including miRNAs known to mediate defense by targeting auxin-responding factors as well as some cassava-specific miRNAs. Some bacteria-repressed miRNAs included families involved in copper regulation as well as families targeting disease resistance genes. Putative transcription factor binding sites (TFBS) were identified in the *MIRNA *genes promoter region and compared to promoter regions in miRNA target genes and protein coding genes, revealing differences between *MIRNA *gene transcriptional regulation and other genes.

**Conclusions:**

Taken together these results suggest that miRNAs in cassava play a role in defense against *Xam*, and that the mechanism is similar to what's known in *Arabidopsis *and involves some of the same families.

## Background

Very succinctly plant-bacteria interactions can be thought as governed at molecular level mainly by three types of proteins: plant PRRs (pathogen recognition receptors), bacterial effectors and plant resistance proteins. PRRs are proteins recognizing highly conserved structures and molecules in microorganisms named MAMP (microbial-associated molecular patterns) and mediate MAMP-triggered immunity (MTI), which is efficient against non-adapted pathogens. Pathogens have developed effector proteins to suppress MTI. In turn, plants can counteract the action of effector by the specific recognition of effectors mediated by resistance proteins which will trigger a strong defence response known as ETI (effector-triggered immunity) [1].

During the past decade, small RNAs have also been found to be key players in mediating plant-pathogen interactions as well as many other biological processes. microRNAs (miRNAs) are important regulators of eukaryotic gene expression. They are transcribed from nuclear *MIRNA *genes by RNA polymerase II (RNA pol II) into primary miRNAs (pri-miRNAs). The pri-miRNAs are then processed in plants by Dicer-like proteins (DCL) into precursor miRNAs (pre-miRNAs) which form a characteristic hairpin-like structure [2,3]. A subsequent processing step by DCL slices the pre-miRNA to form a miRNA:miRNA* duplex (21-22 nt). The duplex is then methylated and exported from the nucleus to the cytoplasm where it is recognized by an argonaute (AGO) protein and incorporated into the RNA-induced silencing complex (RISC). Only the mature miRNA strand (usually the one having less stable 5' end pairing) is retained in the complex, while the passenger (miRNA*) strand becomes degraded [3]. However, in some cases, miRNA* has been detected as being expressed at the same or even at higher levels than the leader strand and may have silencing activity [4]. The RISC complex will guide complementary mRNA (targets) silencing, usually by cleavage between the 10^th ^and 11^th^nt of the paired miRNA [3].

An early miRNA pathway control mechanism comes from *MIRNA *gene transcription regulation by *cis*-regulatory elements and *trans*-acting factors. Recent works have attempted to identify key elements involved in miRNA regulation [5-9]; however, little is yet known about miRNA co-regulation under different conditions and the mechanisms involved.

Most known plant miRNAs target transcription factors which play an important role in regulating plant development. There is now increasing evidence of miRNA's importance in response to biotic and abiotic stress in plants [2,10]. Reprogrammed miRNA-mediated gene expression during plant immune response has not been studied in depth, but is potentially an important element for controlling pathogen invasion. It has been demonstrated that bacteria-induced miR393 mediates anti-bacterial defense of *A. thaliana *against *Pseudomonas syringae *pv. tomato (Pst) by targeting TIR1, an F-box family of auxin receptors and consequently repressing auxin signaling [11]. In turn, bacteria use effector proteins to disrupt miRNA accumulation [12]. The repertoire of known bacterial-responsive miRNAs has increased and includes several families known to regulate hormone signaling, such as miR160, miR167 and miR390 involved in auxin signaling, miR159 involved in ABA signaling and miR319 involved in jasmonic acid signaling [13-15].

Cassava *(Manihot esculenta) *is a staple crop which stores important quantities of starch in its roots. These roots constitute the main source of calories for more than half a billion people around the world, mainly in tropical regions [16]. The starch also has important uses in industry, including bioethanol production [16-18]. Cassava has remarkable tolerance to abiotic stress, it can be cultivated in low-fertility acidic soils and is highly tolerant to drought [16]. Its production can be severely affected by cassava bacterial blight (CBB), caused by gram-negative bacteria *Xanthomonas axonopodis *pv. manihotis *(Xam)*. This disease is present in all regions where cassava is grown and production losses can reach up to 80% or 100% [19]. CBB incidence, as that of many plant diseases, is expected to increase greatly with climate change [20]. This, along with the increasing human population, makes it essential to understand the underlying mechanism of plant antibacterial defense, aimed at producing biotechnological strategies for crop genetic improvement.

The cassava miRNA repertoire is mostly unknown. Up to 20 conserved miRNA families have been indentified in ESTs collections by using bioinformatics approaches [21-23] and the expression of 23 mature miRNA families in cassava and other euphorbiaceous has been studied [24]. However no miRNAs from cassava are currently deposited in miRBase, the consensus database for verified miRNAs [25]. The first draft of the cassava genome was released in October 2009 and a new version was made available in October 2010 [26]. This is an important tool for the discovery and prediction of new and specific cassava miRNAs.

This study characterizes the cassava miRNA repertoire using expression data from small RNA libraries and identifies pre-miRNAs in the cassava genome. The miRNA-mediated response to *Xam *infection in cassava is also analyzed as well as possible transcription factors involved in miRNA regulation.

## Results

### Deep sequencing of cassava sRNA libraries

**Figure 1:**
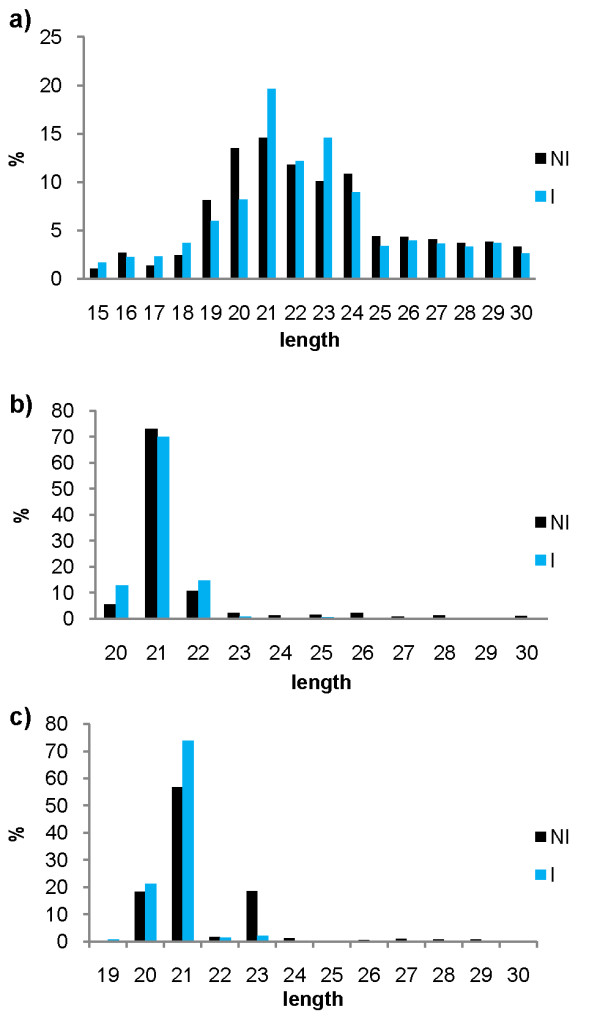
**Size frequencies of trimmed and miRNA-mapped sRNA reads from inoculated and not inoculated cassava libraries**. **a) **Size frequencies of trimmed unmapped reads in both libraries. **b) **Size frequencies of reads in both libraries mapping to known plant miRNAs. **c) **Size frequencies of reads in both libraries mapping to new cassava miRNAs.

Small RNA profiling libraries sequenced with Illumina SBS technology were used to study the role of cassava miRNAs in response to *Xam*. Two small RNA libraries were constructed using RNA extracted from leaves and stems from the *Xam*-resistant cassava variety MBRA685. One of them was not inoculated **(NI) **and the other inoculated **(I) **with the highly aggressive *Xam *strain CIO151. The inoculated library was constructed from an RNA pool of various post-inoculation times so only robust and consistent responses could be detected. 15 and 11 million reads trimmed reads (adapters removed) were obtained from the NI and I libraries, respectively (Table 1). Most trimmed reads in both libraries were in the 20-24nt range (most small RNA sizes) (Figure 1a). Processed and raw files for these libraries can be accessed at the NCBI Gene Expression Omnibus under accession number GSE29379.

**Table 1:** General statistics for cassava small RNA library processing and mapping

	Non-inoculated reads	Inoculated reads
Total	31,434,907	15,968,516

High quality, adaptor-trimmed	15,084,481	11,871,330

Matching cassava genome	8,844,221	7,226,346

Matching cassava genome, Non-redundant (nr)	1,183,868	653,480

Not matching rRNAs, snoRNAs, tRNAs*	5,655,383	5,187,027

Not matching rRNAs, snoRNAs, tRNAs (nr)+	2,906,014	1,664,361

Matching *Xam *genome	45,416 (0.8%)	298,811 (5.2%)

Matching *Xam *genome (nr)	6,952 (0.2%)	80,850 (4.8%)

Matching known miRNAs	42,356 (0.7%)	122,273 (2.3%)

Matching known miRNAs (nr)	4,921 (0.1%)	3,398 (0.2%)

Known miRNA families	53	48

Matching cassava intergenic regions	522,080 (9.2%)	599,178 (11.5%)

Matching cassava intergenic regions (nr)	87,590 (3%)	64,536 (3.8%)

*Percentages and normalized values throughout the work were calculated on these total reads. +percentage for non-redundant reads were calculated based on these total reads

Reads from both libraries were mapped to the cassava genome (Table 1), resulting in eight million (NI) and seven million (I) reads perfectly mapping to the genome. Reads mapping to known snoRNAs, tRNAs, and rRNAs were then removed and 5,655,383 and 5,187,027 reads were obtained from the NI and I libraries, respectively, and used for further analysis.

Both libraries were also mapped against the *Xam *genome to verify the presence of nucleic acids from bacteria in the I but not in the NI library (Table 1). As expected, fewer reads from the NI library mapped the *Xam *genome (0.8%) compared to the I library (5.2%). Most NI reads matching the *Xam *genome also matched the cassava genome (91%) and were thus shared short regions between both genomes. Only 33% of the I reads mapping to the *Xam *genome corresponded to these shared regions; the remaining 67% may represent non-coding RNA or degraded RNA from *Xam*.

### miRNA and pre-miRNA identification

To identify conserved miRNAs, reads from both libraries were mapped to a set of known plant miRNAs from miRBase [25] and PMRD [27]; 56 conserved miRNA families were identified in cassava. Reads mapping to known miRNA were predominantly 21nt long (Figure 1b). These miRNAs were also screened using miRProf [28] and all 56 families were confirmed with this method. Unlike miRBase, PMRD includes miRNAs without experimental validation. Only seven PMRD-exclusive miRNA families mapped against small RNA cassava libraries; these were taken into account for pre-miRNA identification but no precursor could be identified in the cassava genome and were excluded from later analysis.

Reads for the 56 conserved miRNAs families were mapped against the genome and 485 adjacent regions were analyzed finding that 116 met the main criteria for being considered as real pre-miRNAs (structural features and secondary structure statistical test). These pre-miRNAs represented 24 miRNA families (Additional file 1). pre-miRNAs for some families were not found which could be explained by considering the cassava genome incomplete sequencing or the presence of non-canonical pre-miRNAs.

To identify novel miRNAs, reads ranging from 20 to 24nt from both libraries were mapped against cassava intergenic regions (Table 1) and the adjacent ± 150nt region was extracted (in total 64,876 regions). miRchek [29], miPred [30] and miReap [31] were used for initial filtering of possible pre-miRNAs. Candidates meeting the main criteria mentioned above (structural features and secondary structure statistical test), as well as having supporting evidence from miRcheck, miReap or miPred, were considered to be real pre-miRNAs. Twelve new miRNA precursors from 12 miRNA families were identified and named Cass_miRA through Cass_miRL (temporary names) (Additional file 1).

Reads mapping to the predicted novel miRNAs were mostly 21nt long, as was observed for conserved miRNAs (Figure 1c). A Blastn [32] search of Genbank's nucleotide nr database for high pre-miRNA conservation in other plant genomes revealed no similarity (e-value < 0.001, > 80% coverage) with plant nucleotide sequences different from cassava in 10 out of the 12 pre-miRNA families, whereas two pre-miRNAs, Cass-miRK and Cass_miRL, resemble non-coding sequences from *Populus thricocarpa *and *Helianthus petiolari *respectively.

### Target prediction

Targets were predicted for all miRNAs identified among annotated genes in the cassava genome using a modified version of miRanda software [33]; 277 possible targets for 43 conserved miRNA families were identified as well as 70 targets for the 12 new miRNA families (Additional file 2). Targets were also searched for all miRNAs in the cassava genome using psRNAtarget http://plantgrn.noble.org/psRNATarget/. Additional support as real targets was obtained for 162 out of the 347 targets identified with miRanda (Additional file 2). A high percentage of possible targets identified were transcription factors (24%), as is common for many known plant miRNAs [2]. Other commonly predicted targets were kinases (7%), DNA binding proteins (3%) and disease resistance proteins (3%). Four families (miR167, miR397, miR894 and Cass_miRJ) had among their possible targets genes involved in starch biosynthesis or metabolism, as identified by similarity with genes in the KEGG pathway (map00500), which could be important focus points for biotechnological strategies aiming at bioethanol production.

### miRNA differential expression

**Figure 2:**
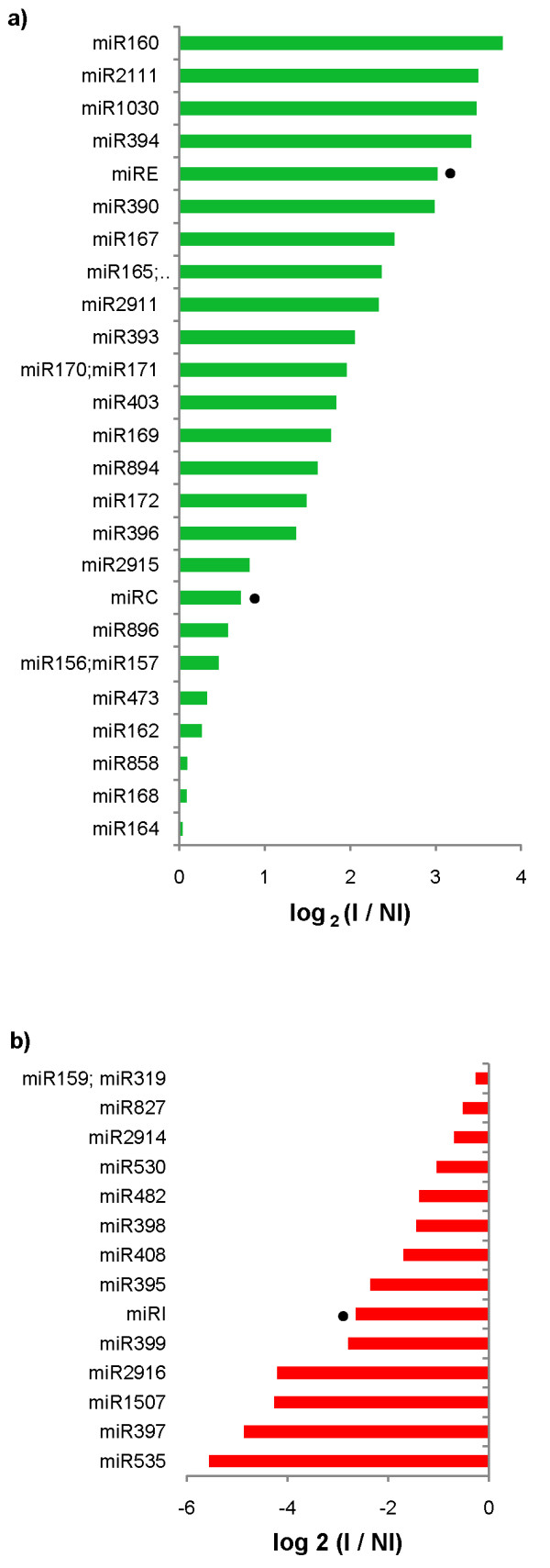
**Differential expression of miRNA families in response to bacterial infection**. a) Bacteria-induced miRNAs. b) Bacteria-repressed miRNAs. Only families having expression values greater than 10 normalized reads in at least one small RNA library are shown. Black circles indicate novel cassava miRNAs.

**Figure 3:**
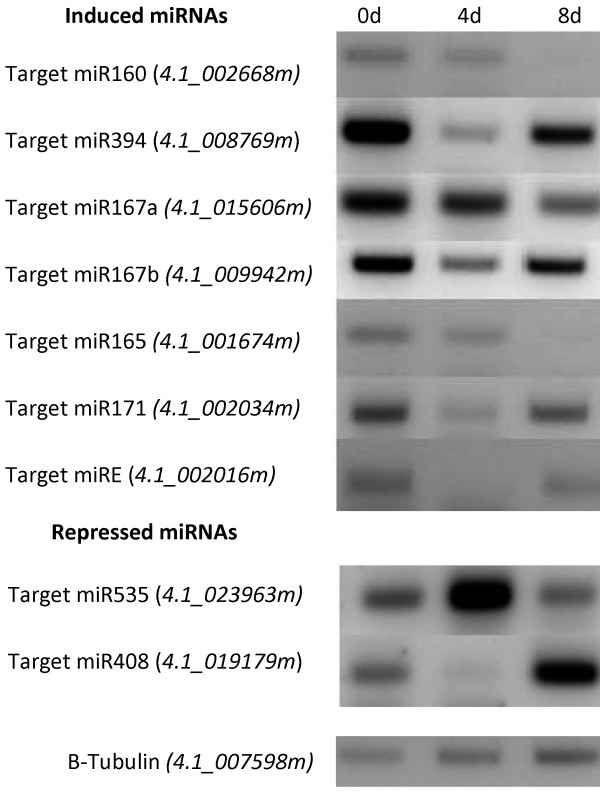
**miRNA target expression shown by semi-quantitative reverse transcription PCR**. RNA from MCOL1522 cassava plants leaves inoculated with Xam CIO151collected at 0 days post inoculation (dpi), 4 dpi and 8 dpi. Gene names from the cassava genome annotation appear in parenthesis.

Cassava's miRNAs expression levels were quantified as normalized reads mapping to each mature miRNA in both libraries. 10 conserved miRNA families had highly increased expression (log_2_fold > 2) in response to *Xam *infection (Figure 2a), including miR160 and miR167 families which are both known to target auxin response factors (ARFs) [13] and miR393 and miR390 families which are also known to regulate auxin signaling [13]. Predicted targets for miR160 in cassava were ARF-like genes whereas predicted targets for miR167 included phosphatases and peptidases. These targets' downregulation in response to bacteria was confirmed through semi-quantitative RT-PCR (Figure 3). It was possible to confirm predicted target downregulation for other induced conserved miRNA families, including miR394, miR165 and miR171 which target an F-box family protein, an ATHB transcription factor and a scarecrow-like transcription factor, respectively (Figure 3). In some of these cases (miR171, miRE, miR394, miR197b) target expression decreased at 4 days post-inoculation (dpi) and then increased at 8 dpi, but never reached higher expression values than those without inoculation. This can be expected since libraries were constructed from an RNA pooled from different times post inoculation. In spite of the importance of these data, a time specific quantification of mature miRNAs is still needed to make an accurate correlation with target expression.

On the other hand seven miRNA families' expression became reduced in response to *Xam *infection (log_2_fold < -2) (Figure 2b). These repressed families included miR535, miR395 and miR482 which were predicted to target various candidate NB-LRR and LRR disease resistance proteins. The miR397, miR398 and miR408 families were also repressed (log_2_fold < -1.4); they are involved in copper regulation by targeting laccases, copper superoxide dismutases and plantacyanins, respectively [34]. It was possible to confirm bacteria-induced increased expression for a resistance-like protein predicted to be targeted by miR535 (induced at 4 dpi) and for a plantacyanin predicted to be targeted by miR408 (induced at 8 dpi) (Figure 3).

Novel miRNAs had overall low expression levels. Only three novel miRNAs (Cass_miRC, Cass_mirE and Cass_miRI) had expression values higher than 10 normalized reads in at least one library. Thus, while many showed differential expression in response to bacteria they were not taken into account due to their low expression values. Species-specific miRNAs are known to have low expression compared to conserved miRNAs [35]. Cass_miRE was highly induced in response to bacteria (log_2_fold = 3) (Figure 2a) and downregulation of its predicted target, a kinase related to *A. thaliana FERONIA*, was determined by RT-PCR (Figure 3). This gene is known to be involved in pathogen interactions and pollen development [36,37].

**Figure 4:**
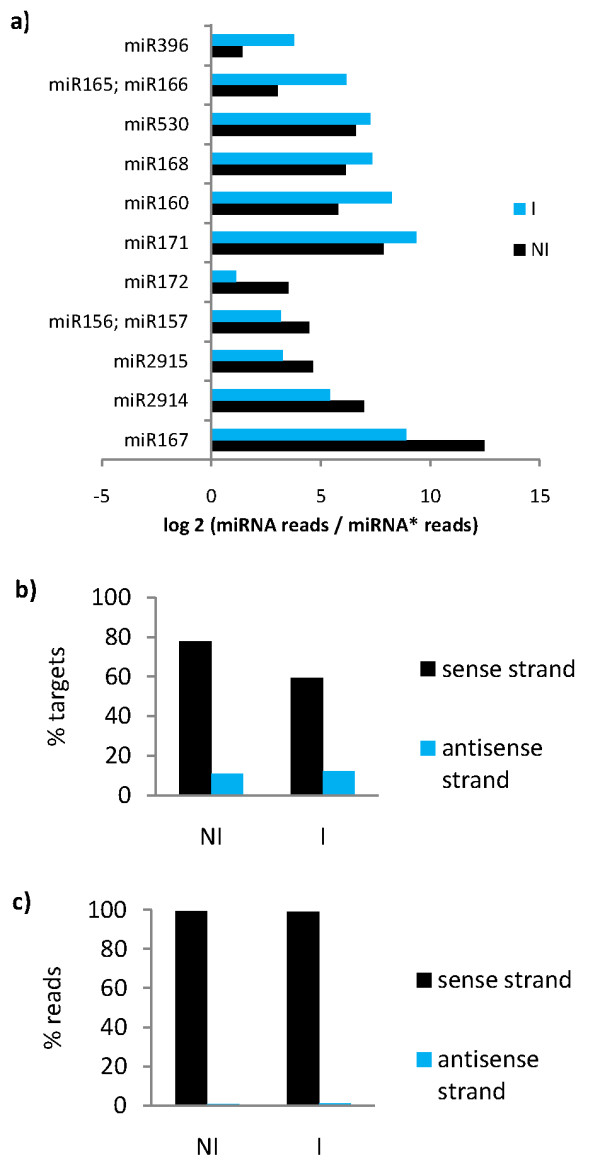
miRNA* expression and transitivity analysis. a) log2 miRNA/miRNA* ratio shown for families in which miRNA* expression was detected. b) Targets for which reads matching sense and antisense strand were detected in both libraries. c) Reads mapping to target mRNA sense and antisense strands in both libraries.

The passenger strand (miRNA*) becomes rapidly degraded after miRNA:miRNA* duplex disruption and leader strand incorporation into RISC [3]. Its expression can nevertheless be detected in small RNA libraries and it is considered supporting criteria for miRNA validation [38]. miRNA* expression for cassava miRNAs was quantified as the number of reads in each library strand-specifically mapping to cassava miRNAs* predicted from pre-miRNAs. miRNA* expression was detected for 11 conserved miRNA families. All families had miRNA/miRNA* ratios higher than 1 in both NI and I libraries, meaning that as expected the leader strand was always more highly expressed than the passenger strand. The miRNA/miRNA* ratio changed for each miRNA family between NI and I library (Figure 4a). A change in miRNA/miRNA* ratio may have indicated differential degradation of the miRNA* strand in different conditions; however, it was most likely a stochastic effect due to the detection of degrading molecules.

It is thought that miRNA silencing signal can be amplified by the production of transient siRNAs derived from cleaved target mRNAs [39]. Reads from both libraries were specifically mapped to predicted cassava miRNA targets' sense or antisense strand to assess the extent to which transitivity or miRNA silencing signal amplification occurred in cassava. Reads mapping to the antisense strand would indicate the presence of dsRNA-derived transitive siRNAs after RdRP recognition, while reads mapping to the sense strand are likely to be predominantly cleaved miRNA target products as well as possible siRNAs. The percentage of predicted miRNA targets with reads mapping to the antisense strand (targets producing transitive siRNAs) in both libraries was around 10% (Figure 4b). Reads mapping to miRNA targets' antisense strand (107 reads in NI, 235 reads in I) were around 1% of reads mapping to predicted targets (Figure 4c), i.e. possible transitive siRNA expression. This showed that only a small percentage of targeted genes produced transitive siRNAs and they were produced in very small amounts compared to miRNA target cleavage rate. These results suggest that miRNA signaling amplification through target transitivity is not a prevalent mechanism in cassava.

### Promoter analysis

**Figure 5:**
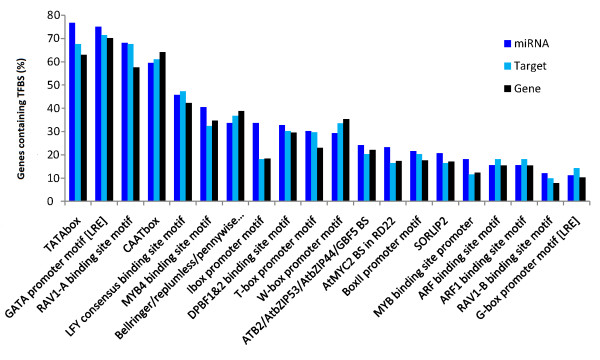
**Transcription factor binding sites frequencies in cassava MIRNA genes, miRNA target and randomly selected protein genes**. Percentage of genes containing at least one binding site for a transcription factor in their promoter regions are shown for each group of genes analyzed.

*MIRNA *genes are transcribed by RNA pol-II after recognition by transcription factors; however, little is known about miRNA transcriptional regulation [5]. Transcription factor binding sites (TFBS) were identified in the 1000nt promoter region upstream of identified cassava pre-miRNAs to find possible patterns in *MIRNA *gene regulation in response to bacteria. Binding sites for 59 transcription factors were identified in 116 miRNA promoter regions. Commonly found regulatory motifs included the TATA and GATA box and RAV (ABI3/VP1-related) and LFY (Leafy) TFBS (Figure 5).

It has been found that ARF binding sites are over-represented in auxin-related miRNA families like miR160 and miR167, thus forming a regulatory loop [40]. In our results miR160 was also the family having the most ARF binding sites identified (10 out of 44 ARF binding sites were found in miR160 promoter regions).

The same predictions were then made in promoter regions of cassava miRNA target genes and a set of 1,000 randomly-chosen cassava genes to determine whether miRNAs were differently transcribed from other groups of genes. Paired wilcoxon tests were made comparing the frequency distributions in each group finding significant differences (α = 0.1) between *MIRNA *genes and randomly selected genes (*p*-value = 0.001248) and between *MIRNA *genes and miRNA targets (*p*-value = 0.08904), whereas no differences were found between target genes and randomly selected genes (*p*-value = 0.5462). However, no significant differences were found between the frequency distributions of miRNA families highly induced or repressed in response to bacteria and those of non-differentially expressed miRNA families (*p*-value > 0.05).

## Discussion

A general model has been proposed based on *Arabidopsis-Pseudomonas *interaction results describing miRNA-mediated response to bacterial infection. In this model miRNAs targeting negative defense response regulators are induced and miRNAs targeting positive regulators (e.g. resistance genes) are repressed upon bacterial infection [41,42]. In this work we reported the differential expression of some cassava miRNA families in response to *Xanthomonas axonopodis *pv. manihotis, generally agreeing with this model. miRNAs induction was found to be involved in regulating auxin signaling: miR160, miR167, miR390 and miR393 [11,13]. Conservation of these miRNA families' expression pattern in *Arabidopsis *and cassava pointed to a plant conserved defense mechanism, likely to be related to PTI, since some of these families have been shown to be induced in response to flagellin [14]. Thus, auxin signaling disruption seems to be an important strategy for impairing bacterial growth in plants. Recent work has shown that enzymatic disruption of auxin signaling is an important mechanism for broad-spectrum resistance and that pathogens secrete auxins during infection to render a plant vulnerable by loosening the cell wall [43,44]. Some conserved miRNA families not present in *A. thaliana *were induced expressed in response to *Xam *infection, including miR2911 and miR1030 families which have so far only been identified in *Populus euphratica *and *Physcomithrella patens*, respectively [45]. The role they might play in mediating plant-bacteria interactions remains unknown.

miRNA downregulation showed in this work also agrees with the general model, since three repressed families (miR535, miR395 and miR482) were predicted to target NB-LRR and LRR resistance-like genes in cassava. These genes' subsequent increased expression may have a role in cassava-specific ETI response; however, this still needs to be demonstrated. Other repressed families were miR397, miR398 and miR408, known to be involved in copper regulation and to be differentially expressed in response to biotic stress [34,46,47]. Copper is widely used as a pesticide in agriculture due to its antibacterial effect [48]. It has been shown that some *Xanthomonas oryzae *strains use transcription activator-like (TAL) effector proteins to modify copper distribution in rice to render the plant susceptible [49]. miRNA-mediated regulation of copper homeostasis could also be crucial as a bacterial defense mechanism.

Transitivity was analyzed by quantifying possible target cleavage-derived transient siRNAs. This mechanism has been studied in detail for some specific cases in *A. thaliana *[50-52] and in the moss *Physcomitrella patens *[53]. A recent study has suggested, however, that the mechanism could be more widespread than once thought; analyzing next-generation sequencing of small RNA libraries, the same percentage was found for small 21nt reads mapped against *Arabidopsis *miRNA targets in both sense and antisense strands [39]. On the contrary, it was found in this work that reads mapping o cassava targets' antisense strand were greatly under-represented in cassava libraries (< 1%) and only around 10% of predicted targets produced antisense reads, agreeing with transitivity being an infrequent mechanism.

Finally, TFBS were predicted on the promoter region of *MIRNA *genes finding that *MIRNA *genes appeared to have overall a different frequency distribution from those of protein coding genes including miRNA targets suggesting the presence of regulatory elements acting specifically on *MIRNA *genes. It has been previously reported that *MIRNA *genes are abundant in TATA-box, AtMYC, ARF, SORLEP3 and LFY binding sites, compared to protein encoding genes [40]. In our results the frequency distribution of TFBS in these groups of genes reveals that namely the TATA-box, MYB4 and L1-box motifs were more abundant in *MIRNA *genes. However, no differences in TFBS frequencies could be found between bacteria-induced or bacteria-repressed miRNA families and families that were not differentially, this suggests that miRNA transcriptional regulation in response to bacteria may be family-specific or loci-specific.

## Conclusions

This work has shown in-depth characterization of cassava miRNAs in response to *Xam *infection and has shed new light on these molecules' importance in plant-pathogen interactions. These data are encouraging but still preliminary and further experimental validation is still needed to fully understand the impact of the miRNA pathway in the cassava-*Xam *interaction. Understanding miRNA regulation and/or that of their targets could (given miRNA pathway flexibility) lead to developing better biotechnological strategies aimed at producing cassava plants having enhanced resistance to pathogens.

## Methods

### Plant materials and plant inoculation

Cassava plants were grown from mature stem cuttings and kept in a greenhouse at 26-30°C, with 12 h day-light photoperiod and 80% relative humidity. Cassava variety MBRA685 (resistant to *Xam*-CIO151) was used for small RNA library construction and variety MCOL1522 (susceptible to *Xam-*CIO151) was employed for RT-PCR experiments.

Six-week-old plants were inoculated with 36 h-old cultures of the aggressive *Xanthomonas axonopodis *pv. manihotis strain CIO151 in both leaves and stems. Leaves were inoculated by piercing six holes in the mesophyll and placing a 5 μL drop of a liquid *Xam*-MgCl_2 _culture calibrated at OD_600 nm _= 0.002 (1 × 10^8^cfu/ml). Two leaflets per leaf and three leaves per plant were inoculated. Stems were inoculated by puncture. At least three plants per collection time were inoculated for each experiment.

### Small RNA library construction and sequencing

For the inoculated library leaves and stems were collected from inoculated plants (0 h post inoculation -hpi, 6 hpi, 24 hpi, 2 days post-inoculation -dpi, 5 dpi, 7 dpi and 15 dpi).. RNA extractions were made using a LiCl-acid phenol:chloroform method. RNA extractions from inoculated plants (at least six plants per time point) were pooled together in equal amounts. For the non-inoculated library a single RNA extraction from tissues from six untreated plants was used.

For library construction, adapters were added to total RNA (150 μg/mL) and cDNA was synthesized using a Superscript Double-Stranded cDNA Synthesis kit (Invitrogen). cDNA was enriched through PCR and the small RNA fraction (10-50 nt) was then separated on a denaturing polyacrylamide gel. Libraries were constructed at the BC Cancer Agency's Michael Smith Genome Sciences Centre http://www.bcgsc.ca/ and sequenced with Illumina SBS deep sequencing technology using an Illumina Genome Analyzer IIx http://www.illumina.com/.

### Analysis of small RNA sequencing data

Various quality filters were applied to raw reads data. Sequences having less than 0.6 chastity value (measured by sequencing software) in the first 25 bases were removed. Adapter sequences were then removed with an in-house C++ program; this program removed any sequence fragments larger than 10nt from the original sequence matching the adapter sequence used in the libraries (5'-ATCTCGTATGCCGTCTTCTGCTTG-3') and sequence fragments shorter than 10nt were only removed if they started after the 15^th^nt in the original sequence. The program used the EMBOSS wordfinder tool [54] to find adapter fragments (parameters: *minimum match score *= 70, *alignment width *= 6, *gap opening *0.0 + *gap extension *10 or *gap opening *500 + *gap extension *10). Sequences shorter than 15nt, as well as low-complexity and low-quality sequences, were then removed using UNIX commands.

Reads were mapped against known snoRNAs, tRNAs and rRNAs obtained from the Rfam Database [55] and then removed. This was done by using Blastn, v. 2.2.21 (parameters:*e-value *< 0.0001, *ungapped, word size *= 4) [56]. Unless indicated, these were the standard parameters used for all mapping analysis.

Reads were then mapped to the cassava genome v. 4 (Cassava Genome Project 2010, http://www.phytozome.net/cassava) (parameters: *e-value *< 1e-5, 100% identity) and the preliminary version *Xanthomonas axonopodis *pv. manihotis genome (*Xam *genome project, unpublished (parameters; *e-value *< 1e-5, 100% identity).

### miRNA and pre-miRNA identification

To identify phylogenetically-conserved miRNAs, reads from both small RNA libraries were mapped to the set of all mature Viridiplantae miRNAs obtained from miRBase release 16, September 2010 [45] and the complete plant miRNA set obtained from the Plant MiRNA Database PMRD, v. September 2010 [22]. Reads having less than two mismatches with a known miRNA were considered conserved miRNAs [38]. Conserved miRNAs (and their expression profiles) were also identified using the miRProf tool from the UEA sRNA toolkit (default parameters) [28]. Reads from both libraries in the 20-24nt size range were mapped on the cassava genome (Cassava Genome Project 2010, http://www.phytozome.net/cassava) to identify possible novel cassava-specific miRNAs.

Pre-miRNA analysis used the adjacent region (-150, +150 nt) to mapped positions from a read of interest, extracted from the genome using fastacmd [56]. These were then mapped onto annotated cassava genes and regions having large overlaps (> 25%) with genes were removed from further analysis.

Two main criteria were considered for real pre-miRNAs: structural features identified from predicted foldings and a secondary structure statistical test. Candidate pre-miRNAs were folded with RNAFold from the Vienna RNA package [57] and mFold v. 3.5 [58]. Additional secondary structures for easier visualization were obtained using the RNAfolding utility in the sRNA toolkit [28]. Structures were analyzed with in-house pearl scripts. Real pre-miRNAs were considered if they had less than six mismatches between predicted mature miRNA and miRNA*, few (maximum three) and short (less than 3nt) asymmetric bulges in the structure [38,59]. Secondary structure minimum folding energy (MFE) significance was calculated using a statistical test; 1,000 random sequences were generated for each possible precursor, maintaining the same base composition and dinucleotide frequencies (k-let = 2) using ushuffle [60]. Random structures were then folded using RNAfold [57] and the p-value was calculated as the percentage of random structures having an MFE equal to or lower than the original precursor [61]. Real pre-miRNAs must had < 0.05 p-value.

Candidate novel pre-miRNAs were analyzed and filtered using miRcheck (default parameters) [29] MIREAP (parameters, B = 55, a = 19, b = 24, u = 1,000, e = -10 kcal/mol, d = 200, *p *= 7, v = 10, s = 100, f = 10) [31]https://sourceforge.net/projects/mireap/ and miPred (random-forest prediction module not used) [30] to obtain additional prediction support.

### miRNA targets prediction

miRNA targets were searched using a modified miRanda version v. 2.0 September 2008 [33], as previously described to meet plant miRNA:target pairings criteria [62]. Targets were also searched using psRNAtarget, v. December 2010 (default parameters, http://plantgrn.noble.org/psRNATarget/), an update to the miRU software [63]. Targets were searched in cassava coding sequences (Cassava Genome Project 2010, http://www.phytozome.net/cassava).

### miRNA, miRNA* and transient siRNAs *in silico *quantification

Reads from both libraries were mapped strand-specifically to quantify miRNA and miRNA* expression using Blastn (*e-value *< 0.0001, 100% identity, *S = 1 *for miRNA and *S = 2 *for miRNA*). Expression values were assigned for each family instead of each loci due to high similarity in mature miRNA sequences. Expression values were normalized using this formula: matching reads/total reads × 1,000,000.

Reads ranging from 20 to 24nt from both sRNA libraries were mapped strand-specifically against all identified possible targets for all cassava miRNAs to quantify possible transient siRNAs and mRNA fragments generated by miRNA-mediated cleavage (*e-value *< 0.0001, 100% identity, *S = 1 *for miRNA and *S = 2 *for miRNA). Only the -100nt, +100nt region immediate to the predicted miRNA target gene cleavage site was used for mapping to avoid mapping to random RNA fragments not generated by miRNA-cleavage. Expression values were normalized as the number of reads mapping to target/total reads.

### Promoter analysis

The 1,000 nt upstream region of identified cassava pre-miRNAs, predicted miRNA targets and randomly-chosen cassava genes were extracted for identifying promoters; only complete and high-quality sequences were extracted. Regions having overlaps with genes were delimited to exclude the gene, unless the overlap was longer than 600 nt; in this case, the promoter region was not used. TFBS were identified in these regions as described in [6].

Wilcoxon paired tests (two-tailed) were made with R version v. 2.12.0 (R Development Team) comparing TFBS frequencies for each desired group of genes obtained as: Number of genes with a given TFBS/Total of genes evaluated.

### Semi-quantitative RT-PCR analysis

RNA was extracted from leaves and stem tissues from cassava MCOL1522 non-inoculated and inoculated (4 dpi and 8 dpi) with *Xam *CIO151, grown as described above, to quantify miRNA target expression. After DNAse I (Fermentas) treatment, cDNA was synthesized using First Strand cDNA Synthesis with oligo dTs (Fermentas). cDNA concentration was normalized after PCR using tubuline primers (F = 5'-GATCCTACTGGGAAGTACATTGG-3', R = 5'-GATCATTCTCCACCAACTGA-3'). Gene-specific primers for predicted miRNA targets were designed covering the predicted cleavage site. PCRs were performed with 32 cycles a 94°C for 30 s, 54-60°C for 30 s and 72°C for 30 s. The primers used for each target are listed in Additional file 3.

## Authors' contributions

ALPQ: Prepared plant materials, processed the sRNA libraries data and drafted the manuscript. AQ: Participated in miRNA predictions and data analyses. OU: Participated in miRNA predictions and data analyses. PV: Carried out the semi-quantitative RT-PCR analysis. CL: Conceived the study, participated in its coordination and helped to draft the manuscript. All authors read and approved the final manuscript.

## Supplementary Material

Additional file 1 **Cassava pre-miRNAs descriptions**. Positions in the genome are based in the Cassava4 version of the genome available at Cassava Genome Project 2010, http://www.phytozome.net/cassava. Folding energies were calculated with Vienna RNAFold. Click here for file

Additional file 2 **Cassava miRNA targets**. miRanda predictions for each miRNA family identified in cassava, predictions were made using annotated cds from the Cassava4 version of the genome available at Cassava Genome Project 2010, http://www.phytozome.net/cassava. Descriptions are based on the available annotations for the most similar *Arabidopsis *or rice gene. Click here for file

Additional file 3 **Primers used for RT-PCR**. Click here for file
